# Recent Advances in g-C_3_N_4_ for the Application of Perovskite Solar Cells

**DOI:** 10.3390/nano12203625

**Published:** 2022-10-16

**Authors:** Jian Yang, Yuhui Ma, Jianping Yang, Wei Liu, Xing’ao Li

**Affiliations:** 1 New Energy Technology Engineering Laboratory of Jiangsu Province, Institute of Advanced Materials, School of Science, Nanjing University of Posts and Telecommunications (NJUPT), Nanjing 210023, China; 2 Department of Mathematics and Physics, Nanjing Institute of Technology, Nanjing 211167, China

**Keywords:** g-C_3_N_4_, perovskite solar cells, additive, surface modifier layer

## Abstract

In this study, graphitic carbon nitride (g-C_3_N_4_) was extensively utilized as an electron transport layer or interfacial buffer layer for simultaneously realizing photoelectric performance and stability improvement of perovskite solar cells (PSCs). This review covers the different g-C_3_N_4_ nanostructures used as additive and surface modifier layers applied to PSCs. In addition, the mechanism of reducing the defect state in PSCs, including improving the crystalline quality of perovskite, passivating the grain boundaries, and tuning the energy level alignment, were also highlighted in this review. Currently, the power conversion efficiency of PSCs based on modified g-C_3_N_4_ has been increased up to 22.13%, and its unique two-dimensional (2D) package structure has enhanced the stability of PSCs, which can remain stable in the dark for over 1500 h. Finally, the potential challenges and perspectives of g-C_3_N_4_ incorporated into perovskite-based optoelectronic devices are also included in this review.

## 1. Introduction

Fossil fuels created a huge amount of pollution in the environment. Solar energy, as one of the main sources of clean and renewable energy, can solve both environmental pollution and energy demand. Perovskite solar cells (PSCs) are a new type of photovoltaic device that can directly convert solar energy into electrical energy [1,2]. Compared with silicon-based solar cells, the manufacturing cost of PSCs is lower, and their photoelectric characteristics are more prominent [3,4,5]. Moreover, the PSCs can be attached on the flexible substrate, significantly expanding its application scenarios [6,7]. Currently, the lab-scale power conversion efficiency (PCE) has been certified to boost efficiency up to 25.5% [8]. For realizing the commercialization of PSCs as soon as possible [9,10], several urgent breakthroughs are required, such as obtaining a higher PCE, longer-term stability, and eco-friendliness [11,12,13,14,15,16,17].

Generally, the structure of PSCs are composed of a hole transport layer (HTL), a perovskite layer, and an electron transport layer (ETL), along with counter electrodes [18,19]. The defect-induced recombination of photo-generated carriers significantly impacts the extraction and transportation of charges in PSCs [20,21,22,23], which severely diminishes the performance of PSCs, in including short-circuit current density (Jsc), PCE, current hysteresis and stability, etc. [24,25]. For PSCs, defects are mainly located at the interfaces of ETL/perovskite/HTL, as well as at the grain boundaries (GBs) of perovskite films [26,27,28]. Many routes have been explored to reduce defects in PSCs, including additive and interface engineering, etc. [29,30,31,32,33,34,35,36,37,38,39,40]. Meanwhile, it is worth noting that the stability and eco-friendliness of the materials used in additive or interface engineering should also be taken into account.

**Figure 1:**
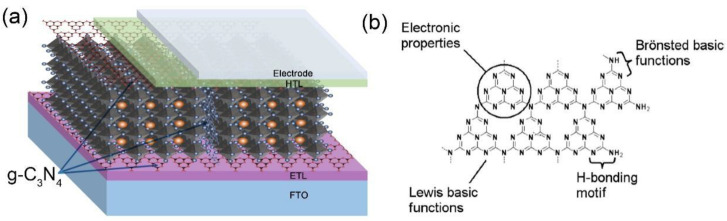
(**a**) g-C_3_N_4_ as an additive or interface engineering material in PSCs, (**b**) surface groups and properties of tri-s-triazine-based g-C_3_N_4_ (reproduced with permission [42]; copyright 2020, American Chemical Society).

Due to the polymeric feature, the surface of g-C_3_N_4_ abounds with N, -NH_2_, -NH-, and other groups, which are facile functionalized by surface modification [41,42,43]. Thus, g-C_3_N_4_ has been utilized in PSCs as an additive or interface engineering material, as depicted in Figure 1a [44,45]. Additionally, different nanostructures (bulk, nanosheets, nanoparticle and quantum dots, etc.) of g-C_3_N_4_ reveal diversified photoelectric characteristics [46,47]. In this work, we summarized the role of g-C_3_N_4_ in PSCs. The main role are as follows: first, facilitating electron transport or perovskite growth via adjusting the energy level or roughness of ETL, corresponding. Second, decreasing the deep electron defect state of PSCs via improving the crystalline quality of perovskite. Moreover, we looked into the development trend of applying g-C_3_N_4_ in perovskite-based optoelectronic devices.

## 2. g-C_3_N_4_ as an Additive in Perovskite Films

g-C_3_N_4_ is a metal–free, non–toxic, and high–yield polymeric semiconductor with an around 2.7 eV bandgap. More importantly, it has excellent thermal and chemical stability [46,48,49,50]. Consequently, g–C_3_N_4_ has been broadly used in pollutant degradation, sensing, optoelectronic devices, and other fields [51,52,53,54,55,56,57,58,59,60]. Figure 1b plotted the molecule structure diagrams of g–C_3_N_4_, based on tri–s–triazine connection patterns. The hexagon triazine ring is comprised of sp^2^ hybridized N and C atoms, with hydrogen bonds between the –NH_2_ groups and the N edge atoms, and they are linked at the end with a C–N bond, creating an extended network–like planar structure.

### 2.1. Pure g–C_3_N_4_ Nanosheets as an Additive

**Figure 2:**
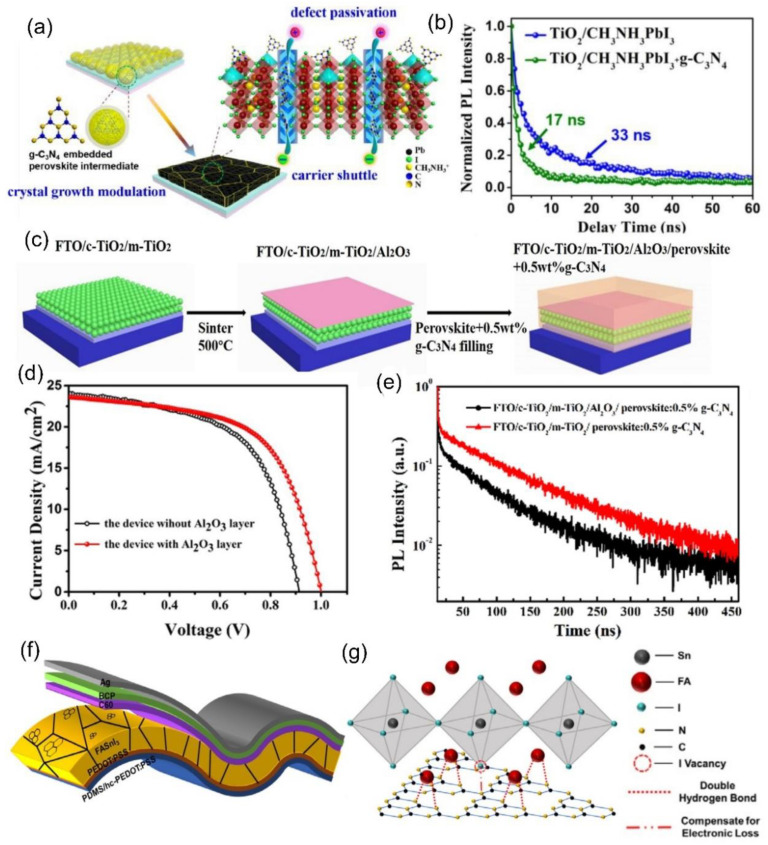
(**a**) Schematic illustration of the functions of g–C_3_N_4_ as an additive in perovskite films. (**b**) TRPL lifetime of g–C_3_N_4_:CH_3_NH_3_PbI_3_ and CH_3_NH_3_PbI_3_ films (reproduced with permission [64]; copyright 2019, Royal Society of Chemistry). (**c**) Fabrication procedure of a device with FTO/c–TiO_2_/m–TiO_2_/Al_2_O_3_/g–C_3_N_4_:CH_3_NH_3_PbI_3_/carbon. (**d**) J–V curves of the devices and (**e**) TRPL spectra of perovskite films (reproduced with permission [68]; copyright 2019, Elsevier). (**f**) Device configuration: PDMS/hc–PEDOT:PSS/PEDOT:PSS/FASnI_3_/C_60_/BCP/Ag. (**g**) Illustration of bonding and passivation between FASnI_3_ and g–C_3_N_4_ (reproduced with permission [72]; copyright 2021, John Wiley and Sons).

g–C_3_N_4_ nanosheets are π–conjugated nanomaterials with two–dimensional structure, and a larger specific surface area, thus conducting the separation of photo–generated charges [61,62]. In 2018, Jiang et al. reported pure g–C_3_N_4_ nanosheets mixed into a perovskite precursor solution as additives [63], suppressing nucleation and slowing down the growth of perovskite during the crystallization process; this results in the g–C_3_N_4_:CH_3_NH_3_PbI_3_ films have larger grain sizes and a lower defect density (n_t_). In 2019, Liao et al. used a method similar to that of Jiang et al. to add g-C_3_N_4_ nanosheets into perovskite films [64]. Importantly, they revealed the location of g-C_3_N_4_ in perovskite. Meanwhile, the mechanisms of defect passivation and charge extraction by g-C_3_N_4_ were clarified. From Figure 2a, it can be seen that g-C_3_N_4_ was uniformly anchored at the surface of the GBs, and the dangling Pb^2+^ can coordinate with the N atom in g-C_3_N_4_, retarding the crystallization of perovskite [65,66]. Moreover, the conductive g-C_3_N_4_ network condensed at the GBs can act as an efficient carrier shuttle, facilitating the electron transport. From the TRPL spectra, as presented in Figure 2b, for g-C_3_N_4_ additive CH_3_NH_3_PbI_3_ films, the PL lifetime reduce to 17 ns, which is less than that of the CH_3_NH_3_PbI_3_ films, indicating ultrafast photo–excited carrier transport due to the addition of g–C_3_N_4_ [67]. 

Yang et al. added g–C_3_N_4_ into carbon-based PSCs [68]. Additionally, the insulating layer was prepared on the surface of the ETL by spin-coating Al_2_O_3_ [69,70,71]. Figure 2c presents the fabrication procedure of the device. From the J-V curve, as shown in Figure 2d, it can be noted that the Jsc has barely changed after incorporating the Al_2_O_3_ layer, which suggests that the conductive g-C_3_N_4_ network at the GBs can provide electrons via a carrier shuttle. This can be proved from the TRPL spectra, as shown in Figure 2e. In the field of wearable electronics, g-C_3_N_4_ nanosheets were applied into flexible tin-based PSCs [72]. Figure 2f plots the device configuration: PDMS/hc-PEDOT:PSS/PEDOT:PSS/FASnI_3_/C_60_/BCP/Ag. The network structure of g-C_3_N_4_ indicates a better lattice match with formamidine cation, which is more conducive to the crystallization of FASnI_3_ films. The illustration of bonding and passivation between FASnI_3_ and g-C_3_N_4_ is shown in Figure 2g. Finally, the stabilized PCE of 8.56% was obtained.

### 2.2. Functionalized g–C_3_N_4_ Nanosheets as an Additive

The pure g–C_3_N_4_ nanosheets tend to agglomerate in the organic or aqueous environment due to the robust van der Waals interactions, resulting in lower dispersibility [49,73]. However, the functionalized g–C_3_N_4_ nanosheets show good dispersity in liquid, mainly caused by the electrostatic repulsion of the charged groups [42]. Here, there are two main methods to achieve functionalized g–C_3_N_4_ nanosheets—doped, and surface modified [74,75].

**Figure 3:**
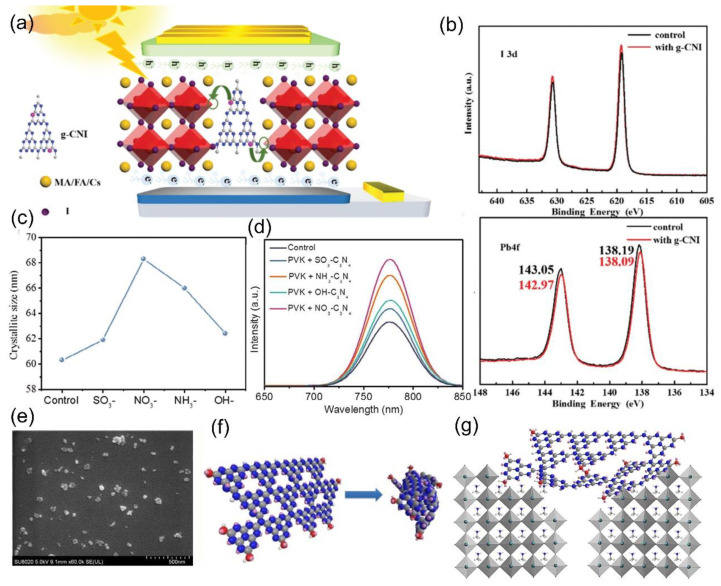
(**a**) Mechanism illustration of g–CNI modified PSCs. (**b**) XPS spectra of Pb 4f and I 3d in perovskite (reproduced with permission [76]; copyright 2019, Royal Society of Chemistry). (**c**) Average crystallite size. (**d**) PL spectra of perovskite films with different functionalized g–C_3_N_4_ (reproduced with permission [41]; copyright 2019, John Wiley and Sons). (**e**) FESEM images of U–g–C_3_N_4_ nanoparticles. (**f**) Scheme of g–C_3_N_4_ fragments coils into U–g–C_3_N_4_ nanoparticles. (**g**) Scheme of the U–g–C_3_N_4_ self–recognizing grain boundaries of CH_3_NH_3_PbI_3_ films (reproduced with permission [82]; copyright 2019, Elsevier).

In 2019, Cao et al. reported that iodine–doped g–C_3_N_4_ (g–CNI) was added into triple cation perovskite films as an additive [76]. Due to the fact that doped iodine can coordinate with dangling Pb^2+^ at GBs, the trap states in PSCs were effectively passivated [77,78]. Therefore, they achieved high–quality perovskite films with fewer trap states [65,79,80]. Figure 3a displays the mechanism of g–CNI modified PSCs. From the XPS spectra, as shown in Figure 3b, for perovskite with g–CNI, the I 3d signal is higher than that of the ref., suggesting the incorporation of g–CNI into the perovskite [81]. After adding g–CNI, the n_t_ reduced to 1.07×10^16^ cm^3^ from 1.43 × 10^16^ cm^3^, with the maximum PCE of up to 18.28%.

Li et al. reported that surface–modified g–C_3_N_4_ with various organic groups (–NO_3_, –NH_3_, –SO_3_ and –OH) was mixed into perovskite films [41], which can improve crystalline quality and passivate defects state at the GBs. Specifically, for NO_3_–C_3_N_4_–based perovskite, the average crystallite size of up to 68 nm was achieved, as exhibited in Figure 3c, leading to a decent photovoltaic performance, which can be proved by the PL spectra, as shown in Figure 3d. It is worth noting that the PL intensity of NO_3_-C_3_N_4_–based perovskite film is the strongest compared to the other perovskite films. This can be ascribed to the better crystallinity of perovskite with the NO_3_–C_3_N_4_ addition, as well as a reduction in the trap states density. As a result, for p–i–n PSCs based on NO_3_–C_3_N_4_, the best PCE obtained was up to 20.08%.

### 2.3. Ultrafine g–C_3_N_4_ Nanoparticles as an Additive

In 2019, Liu et al. reported that g–C_3_N_4_ nanoparticles were introduced into perovskite films as an additive [82]. Here, the ultrafine size of g–C_3_N_4_ nanoparticles is about 20–50 nm, which were successfully synthesized with exfoliated g–C_3_N_4_ nanosheets. The surface of ultrafine g–C_3_N_4_ nanoparticles (U–g–C_3_N_4_) is rich in O–H or N–H groups, which can easily bond with N–H bonds on CH_3_NH_3_PbI_3_ GBs [83]. Thus, it can self–recognize GBs and adhere to them, decreasing the deep electron trap state. The FESEM images of U–g–C_3_N_4_ nanoparticles are plotted in Figure 3e. Figure 3f presents the scheme of U–g–C_3_N_4_ nanoparticles from g–C_3_N_4_ fragments. Figure 3g describes the scheme of the U–g–C_3_N_4_ self–recognizing CH_3_NH_3_PbI_3_ GBs. Finally, the champion PCE of U–g–C_3_N_4_ based planar PSCs is up to 15.8%.

## 3. g–C_3_N_4_ as a Surface Modifier Layer

### 3.1. g–C_3_N_4_ Quantum Dots (g–CNQD) as Modifier Layer

**Figure 4:**
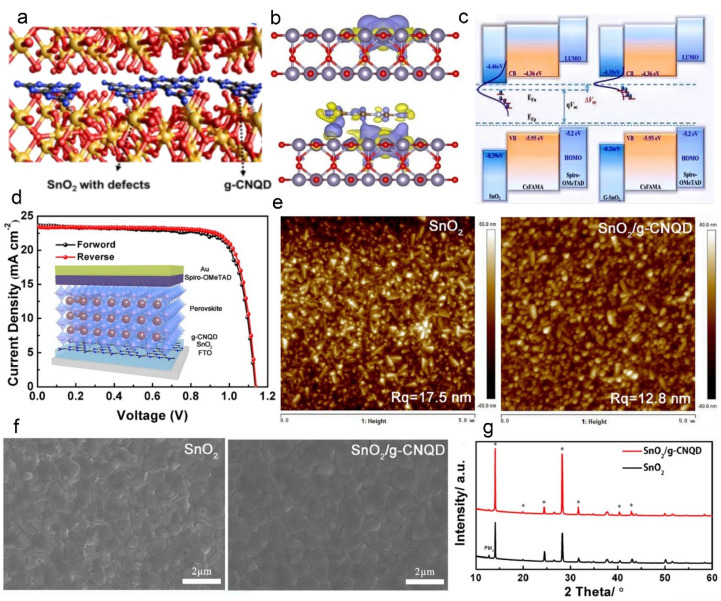
(**a**) The position of G–CNQD in SnO_2_. (**b**) The side view for the charge density difference of SnO_2_ (above) and G–SnO_2_ (below) with oxygen vacancy; the yellow and cyan areas indicate electron depletion and accumulation, respectively. (**c**) Energy band alignment of the devices (reproduced with permission [86]; copyright 2020, Royal Society of Chemistry). (**d**) Schematic illustration of the devices with a g–CNQD layer and a J–V curve. (**e**) AFM images of pristine SnO_2_ film and SnO_2_/g–CNQD films. (**f**) SEM images and (**g**) XRD spectra of perovskite films based on different ETL. The asterisks indicate the main peaks of perovskites structure (reproduced with permission [91]; copyright 2020, Elsevier).

g–C_3_N_4_ quantum dots are a type of zero–dimensional nanomaterial in which electrons and holes cannot move freely [84]. The tiny particle size creates its unique size effect, macroscopic quantum tunnel effect, edge effect [85], etc. In 2020, Chen et al. prepared g–CNQD via acid etching and hydrothermal cure [86]; the diameter of g–CNQD were about 5–10 nm, and they were added into an SnO_2_ colloid precursor, forming nanocomposite ETL (G–SnO_2_). Figure 4a exhibits the position of g–CNQD in SnO_2_. Tiny and conductive g–CNQD could reorganize the electronic density distribution of SnO_2_. The charge density difference between G–SnO_2_ and SnO_2_ is displayed in Figure 4b, which was obtained using the density functional theory. It can be seen that the vacancies surrounding the three Sn atoms interact with g–C_3_N_4_, and an obvious charge redistribution occurs around the oxygen vacancy, resulting in the elimination of the trap state defects [87]. Importantly, g–CNQD can effectively adjust the Fermi level of ETL, promoting electron transport [88,89]. The energy–level diagrams are shown in Figure 4c. For G–SnO_2_ based planar PSCs, the PCE is up to 22.13% with a Voc of 1.176 V. They also exhibit excellent long–term stability under ambient conditions, e.g., about 60% humidity [90].

Liu et al. published a similar report on g–CNQD. The g-CNQD was synthesized with urea and sodium citrate [91], the diameter was about 10–30 nm, and it was well monodispersed. In this work, g–CNQD were intercalated into the ETL/perovskite layer, which facilitates the formation of high–quality perovskite films due to the smoother surface of ETL [92,93]. Figure 4d presents the schematic illustration of the g–CNQD–based device. The roughness of the ETL was evaluated by AFM, as shown in Figure 4e. After intercalating g–CNQD, the root mean square roughness reduced from 17.5 nm to 12.8 nm, suggesting that a smoother ETL was obtained, which is conducive to perovskite growth [94]. From the SEM images and XRD spectra of the perovskite films as shown in Figure 4f,g, it can be seen that the g–CNQD based perovskite films have purer phases, fewer GBs, and lower trap states. Finally, the maximum PCE is noted, up to 21.23% under full air–processing, and without apparent current hysteresis.

### 3.2. g–C_3_N_4_ Nanosheets as a Modified Layer

**Figure 5:**
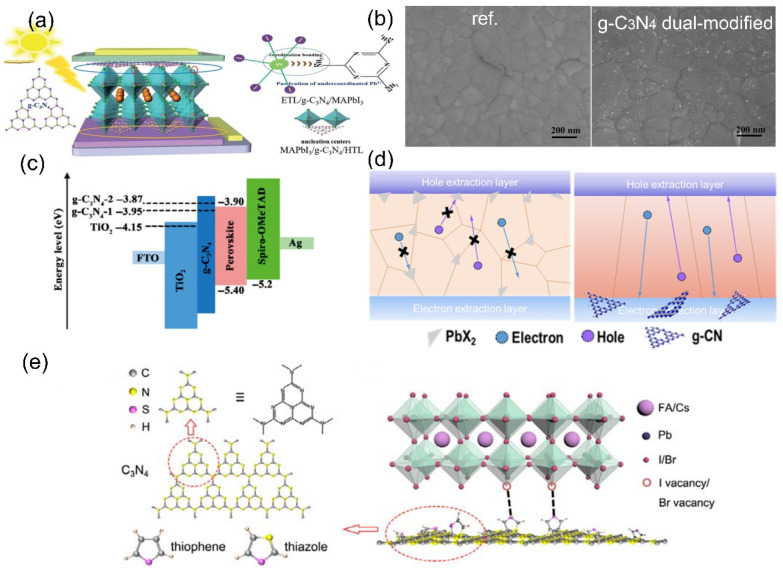
(**a**) Schematic diagram of g–C_3_N_4_ dual–modified PSCs. (**b**) SEM of perovskite films (reproduced with permission [95]; copyright 2018, Royal Society of Chemistry). (**c**) Energy band alignment of the device. The E_F_ level of the ETL, without and with g–C_3_N_4_, are represented with a dotted line (reproduced with permission [98]; copyright 2021, Springer Nature). (**d**) Schematic of the vertical carrier transportation of the perovskite films via buried manipulation (reproduced with permission [99]; copyright 2021, John Wiley and Sons). (**e**) Schematic illustration of the possible interface defect sites (reproduced with permission [100]; copyright 2021, John Wiley and Sons).

In 2020, Liu et al. used multilayer g–C_3_N_4_ to simultaneously modify the upper and lower interfaces of perovskite [95]. For the perovskite/HTL interface, the dangling Pb^2+^ can coordinate with lone–pair electrons on g–C_3_N_4_, reducing the defects state at the perovskite film surface. For the ETL/perovskite interface, the Gibbs free energy of SnO_2_ surface was decreased, which facilitates the preparation of flat and non–pinhole perovskite films [96,97]. Figure 5a displays the schematic diagram of dual–modified PSCs with g–C_3_N_4_. The improvement of perovskite films can be seen from SEM, as shown in Figure 5b. A maximum PCE of 19.67% was obtained for planar PSCs with longer–term stability. The main reason for this was that g–C_3_N_4_ could reduce the surface defects of perovskite films, thus decreasing the migration of iodide and ion mobilization within the perovskite lattices. In 2021, Yang et al. adopted g–C_3_N_4_ nanosheets as a modified layer [98], and the work function of ETL was finely tuned, as shown in Figure 5c, resulting in the enhancement of Voc from 1.01 to 1.11 V, and diminishing the current hysteresis of PSCs. Therefore, the maximum PCE was boosted to 19.55% from its initial 15.81%. Yang et al. reported new buried layers for efficient perovskite [99], which are composed of a mixture of g–C_3_N_4_ and SnO_2_. Due to the fact that amine–rich g–C_3_N_4_ can promote the prenucleation of the Pb–related intermediates, the vertical crystallization of perovskite films were obviously optimized, exhibiting superior carrier transmission characteristics. Figure 5d presents the schematic illustration of vertical carrier transportation via buried manipulation.

### 3.3. Functionalized g–C_3_N_4_ as a Modified Layer

In 2019, Cruz et al. prepared thiazole–modified g–C_3_N_4_ via exfoliation treatment [101], then intercalated it in p–i–n PSCs as ETL. The charge recombination in the interface was suppressed due to the enhanced energy level of electronic interface [88]. Finally, the Voc of 1.09 V and the Jsc of 20.17 mA/cm^2^ were achieved. In 2021, Wang et al. prepared the functionalized g–C_3_N_4_ with thiophene or thiazole by thermal treatment [100]. Then, the functionalized g–C_3_N_4_ were intercalated between ETL and the perovskite layer. Notably, there was a well–matched energy level in the device. Due to the strong chemical affinity between Pb^2+^ and N or S atoms [67], the defect state in the device was efficiently passivated. Figure 5e shows the possible interface defect sites in perovskite, as well as the passivation of thiophene or thiazole. For the thiazole g–C_3_N_4_based device, the maximum PCE increased to 19.23% from pristine 13.42%, with the Voc increasing from 1.02 V. to 1.11 V.

## 4. Conclusions and Future Perspectives

In this paper, we summarize the recent progress of g–C_3_N_4_ application in PSCs. g–C_3_N_4_ is an eco–friendly polymeric semiconductor with a suitable bandgap. Recently, it has been widely applied in PSCs, which can reduce defect states in PSCs, and simultaneously enhance the PCE and long–term stability of PSCs. Specifically, different nanostructures of g–C_3_N_4_ (i.e., nanosheets, nanoparticles, and QDs) used as additive and surface modifier layers have been discussed in detail. The performance of the devices is listed in Table 1.

Pure g–C_3_N_4_ nanosheets are a kind of two–dimensional nanomaterial with large N, –NH_2_, –NH, and other groups, and the N atom in g–C_3_N_4_ can coordinate a bond with dangling Pb^2+^, increasing the perovskite grain size. Moreover, when it functions as an additive, the conductive g–C_3_N_4_ network acts as an efficient carrier shuttle, facilitating electron transport. g–C_3_N_4_ nanoparticles are formed by the self–coiling of the exfoliated g–C_3_N_4_ nanosheets, with an ultra–fine size (20–50 nm), and the surface of the g–C_3_N_4_ nanoparticles contains abundant O–H or N–H groups. Therefore, g–C_3_N_4_ nanoparticles can magically self–recognize GBs in perovskite films, decreasing the deep electron trap state of PSCs. Functionalized g–C_3_N_4_ nanosheets can also be achieved from nanosheets, but the former shows better dispersity in organic solvent. They exhibit a similar effect as additives. Specifically, when used as a modifier layer, they can adjust the energy level alignment of PSCs, making the electron transport more efficient. g–CNQD has a tiny particle size, which can adjust the energy level or roughness of the ETL, facilitating electron transport or perovskite growth, corresponding. These features give the PSCs potential for consideration as next–generation photovoltaic devices, as the most promising means of simultaneously solving environmental pollution and energy demand. The passivation of the perovskite layer is mainly used to improve the PCE and the long–term stability of PSCs. g–C_3_N_4_ can be added in different solutions to prepare efficient PSCs with long–term stability, which have the potential to compete with other conventional silicon cells in the future. Meanwhile, g–C_3_N_4_ shows great promise in solving external and internal concerns, including packaging, additional technology, and reducing charge recombination. Thus, the g–C_3_N_4_ can increase the grain size of perovskite and passivate the interface defect, making it more conducive to charge extraction. However, the development of the g–C_3_N_4_ nanostructure is still in its early stages. Additional methods for improving the efficiency and stability of PSCs require further exploration. We expect to develop new additives exhibiting eco–friendliness, long–term stability, and compatibility with flexible substrates, as well as other new strategies to improve the performance of perovskite. This effort should be closely connected to application of dedicated defect passivation strategies to produce high–performance and enduring stable PSCs.

## Figures and Tables

**Table 1:** Recent development of g–C_3_N_4_ based PSCs photovoltaic performance.

Structure	PCE (%)	Voc (V)	Jsc (mA·cm^−2^)	FF (%)	Ref.	Year
ITO/PTAA/NO_3_-g-C_3_N_4_:CsFAMAPbI_3−x_Br_x_/PCBM/BCP/Ag	20.08	1.11	22.84	79.20	[41]	2019
FTO/c-TiO_2_/g-C_3_N_4_:MAPbI_3_/spiro-OMeTAD /MoO_3_/Ag	19.49	1.07	24.31	74.0	[63]	2018
FTO/c-TiO_2_/g-C_3_N_4_:MAPbI_3_/spiro-OMeTAD /Au	21.10	1.16	23.00	79.0	[64]	2019
FTO/c-TiO_2_/m-TiO_2_/g-C_3_N_4_:CsPbBr_3_/carbon	8.00	1.277	7.80	80.32	[66]	2021
FTO/c-TiO_2_/m-TiO_2_/Al_2_O_3_/g-C_3_N_4_:MAPbI_3_/carbon	14.34	1.00	23.80	60.1	[68]	2019
PDMS/hc-PEDOT:PSS/PEDOT:PSS/g-C_3_N_4_:FASnI_3_/C_60_/BCP/Ag	8.56	0.621	20.68	66.68	[72]	2021
FTO/TiO_2_/G-CNI:CsFAMAPbI_3−x_Br_x_/spiro-OMeTAD/Au	18.28	1.07	22.97	74.0	[76]	2019
FTO/c-TiO_2_/U-g-C_3_N_4_:MAPbI_3_/spiro-OMeTAD/Au	15.80	1.10	23.20	62.0	[82]	2019
ITO/CNQDs:SnO_2_/CsFAMAPbI_3−x_Br_x_/Spiro-MeOTAD/Au	22.13	1.18	24.03	78.3	[86]	2020
FTO/SnO_2_/CNQDs/(FA/MA/Cs)PbI_3−(x+y)_Br_x_Cl_y_/spiro-OMeTAD/Au	21.23	1.14	23.39	79.6	[91]	2020
FTO/SnO_2_/g-C_3_N_4_/MAPbI_3_/g-C_3_N_4_/Sspiro-OMeTAD/Au	19.67	1.14	21.45	80.7	[95]	2020
FTO/c-TiO_2_/m-TiO_2_/g-C_3_N_4_ nanosheets/MAPbI_3_/ Carbon	11.37	1.02	16.91	66	[97]	2021
FTO/c-TiO_2_/g-C_3_N_4_/MAPbI_3_/spiro-OMeTAD/Ag	19.55	1.11	23.69	74.0	[98]	2021
ITO/g-C_3_N_4_:SnO_2_/FA_0.85_MA_0.11_Cs_0.04_PbI_2.67_Br_0.33_·xPbI_2_/spiro-MeOTAD/Au	21.54	1.19	23.21	78	[99]	2021
ITO/PTAA/MAPbI_3_/PC_60_BM/CMB-vTA/AZO/Ag	17.15	1.09	20.17	78.03	[100]	2019
FTO/TiO_2_/thiazole-C_3_N_4_/(FAPbI_3_)_0.875_(CsPbBr_3_)_0.125_/spiro-OMeTAD/Ag	19.23	1.11	22.50	77	[101]	2021

## Data Availability

No new data were created or analyzed in this study. Data sharing is not applicable to this article.
